# The Effect of Chitosan Derivatives on the Compaction and Tension Generation of the Fibroblast-populated Collagen Matrix

**DOI:** 10.3390/molecules24152713

**Published:** 2019-07-26

**Authors:** K. Tu Doan, Pratiksha Kshetri, Natthapume Attamakulsri, Derek R. Newsome, Feifan Zhou, Cynthia K. Murray, Wei R. Chen, Gang Xu, Melville B. Vaughan

**Affiliations:** 1 Center for Interdisciplinary Biomedical Education and Research (CIBER), College of Mathematics and Science, University of Central Oklahoma, 100 N. University Drive, Edmond, OK 73034, USA; 2 Department of Biology, College of Mathematics and Science, University of Central Oklahoma, 100 N. University Drive, Edmond, OK 73034, USA; 3 Department of Engineering and Physics, College of Mathematics and Science, University of Central Oklahoma, 100 N. University Drive, Edmond, OK 73034, USA; 4 Department of Mathematics and Statistics, College of Mathematics and Science, University of Central Oklahoma, 100 N. University Drive, Edmond, OK 73034, USA

**Keywords:** chitosan, *N*-dihydrogalactochitosan (GC), myofibroblast, collagen matrix contraction, optical coherence tomography, Dupuytren’s Contracture, fibrosis

## Abstract

Fibrotic diseases, such as Dupuytren’s contracture (DC), involve excess scar tissue formation. The differentiation of fibroblasts into myofibroblasts is a significant mechanism in DC, as it generates tissue contraction in areas without wound openings, leading to the deposition of scar tissue, and eventually flexing one or more fingers in a restrictive fashion. Additionally, DC has a high recurrence rate. Previously, we showed that *N*-dihydrogalactochitosan (GC), an immunostimulant, inhibited myofibroblast differentiation in a DC fibroblast culture. Our goal of this study was to expand our previous study to include other DC and normal cell lines and other chitosan derivatives (GC and single-walled carbon nanotube-conjugated GC) to determine the specific mechanism of inhibition. Derivative-incorporated and vehicle control (water) anchored fibroblast-populated collagen matrices (aFPCM) were used to monitor compaction (anchored matrix height reduction) using microscopy and optical coherence tomography (OCT) for six days. Fibroblasts were unable to compact chitosan derivative aFPCM to the same extent as vehicle control aFPCM in repeated experiments. Similarly, chitosan derivative aFPCM contracted less than control aFPCM when released from anchorage. Proliferative myofibroblasts were identified by the presence of alpha smooth muscle actin via myofibroblast proliferative assay. In all tested conditions, a small percentage of myofibroblasts and proliferative cells were present. However, when aFPCM were treated with transforming growth factor-beta 1 (TGF-β1), all tested samples demonstrated increased myofibroblasts, proliferation, compaction, and contraction. Although compaction and contraction were reduced, there was sufficient tension present in the chitosan derivative aFPCM to allow exogenous stimulation of the myofibroblast phenotype.

## 1. Introduction

During wound healing, fibroblasts organize connective tissues and synthesize new extracellular matrices (ECMs). These cells migrate to the wound surface and start the secretion and organization of collagen to form granulation tissue [1]. Increased mechanical tension generated via the granulation phase stimulates fibroblast differentiation into myofibroblasts enhanced with cytoplasmic alpha smooth muscle actin stress fibers [2]. When stained, these stress fibers clearly delineate myofibroblasts from fibroblasts [2,3]. The focus of this study is to evaluate the contractile role myofibroblasts contribute to wound contraction and ultimately wound healing. Dupuytren’s contracture (DC), identified by abnormal thickening of palmer fascia, causes a significant decrease in digit mobility [4]. Myofibroblasts, when invading areas where there are no wound openings, facilitate the deposition of unwanted collagenous tissue. The progression of DC is categorized into 4 stages based on the presence of a nodule and degree of digital flexion deformity [5]. DC can be treated via surgery, but due to the high recurrence rate [6,7], repeat surgeries are often needed. The need to investigate alternative therapy for DC is evident.

*N*-dihydrogalactochitosan or glycated chitosan (GC) is a polysaccharide derived from chitosan and is capable of stimulating an immune response [8]. The attachment of galactose to chitosan causes the newly synthesized compound, GC, to be water-soluble [9,10]. This attribute of GC allows for a wide variety of biomedical applications [11]. When GC is injected at the site of scarring, immune cells will recognize GC and trigger an immune response [8]. However, the immune cells will not attack the fibroblasts because they are normal cells that are produced excessively in unnecessary areas. Contemporary researchers are using GC with a combination of phototherapy to treat metastatic tumors [12,13]. It was also found that GC could inhibit cell motility and invasion both in vivo and in vitro [14,15]. Single-walled carbon nanotubes (SWNT) have been shown to shuttle various substances across the cellular membrane without cellular cytotoxicity [16]. GC conjugated with SWNT (SWNT-GC) can get inside the cells and inhibit their migratory properties [17]. Results from past cancer cell studies suggest matrix compaction (defined as lattice height reduction) would be inhibited when treated with chitosan, GC, or SWNT-GC compared to a vehicle control or SWNT-polyethylene glycol (PEG) (SWNT conjugated to an inert compound) [18]. Previous work has shown that chitosan inhibited fibroblast migration [19] and compaction of collagen matrices [20]. Our goal in this study is to investigate whether chitosan and its derivatives GC and SWNT-GC generate similar effects on human dermal fibroblasts and DC cell lines via inhibiting compaction, contraction, proliferation and differentiation.

To test our hypotheses we used the anchored fibroblast-populated collagen matrix (aFPCM) 3D model. DC cells contract the matrix released from anchorage after sufficient tension is generated during compaction [21]. The reduced height of the matrix leading to compaction over time is correlated to the amount of tension generated, leading to contraction [22]. Previous studies from our lab suggest that aFPCM generate maximum tension on or around day 6. Most experiments from this study were carried out during the 6-day period. The height of aFPCM for each treatment group was measured every day and replicates were anchor-released for contraction measurements or staining for identification of myofibroblasts on day 6. Further culture in the presence or absence of transforming growth factor beta-one (TGF-β1) was employed to determine its effects at maximum tension.

## 2. Results

### 2.1. Compaction Analysis of aFPCM 

#### 2.1.1. Chitosan Derivatives, When Incorporated Into aFPCM, Reduce Their Compaction 

**Figure 1:**
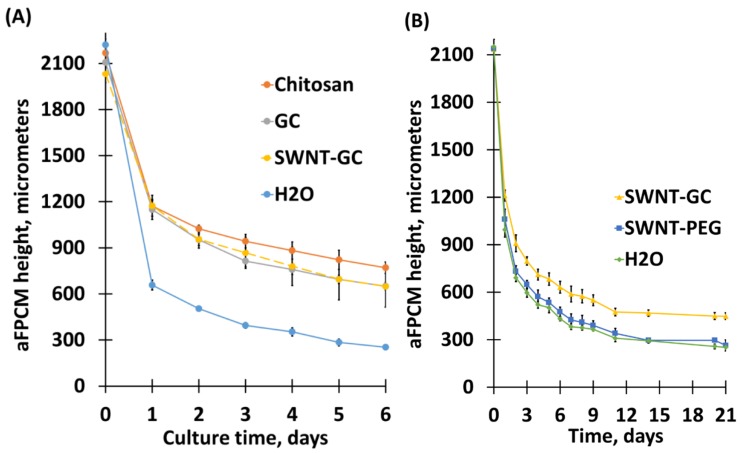
(**A**) Chitosan (C) derivatives reduced compaction of aFPCM over a 6-day period. Cells were cast in aFPCM incorporated with either water, C, GC, or SWNT-GC. Compaction was measured once daily for 6 days. The control (H_2_O) means were significantly different (SD) (*p* < 0.05) than C and GC means at days 1–7. (**B**) SWNT-PEG did not reduce compaction of aFCPM. The control means were SD than SWNT-GC means at days 1–7. SWNT-PEG means were SD than control means only for days 3, 4, and 6. Additional experiments with other cell types demonstrated similar results.

Compaction, defined as aFPCM height reduction, was measured using a Zeiss Primovert inverted light microscope [22]. Previous studies demonstrated a correlation between compaction and tension generation within the matrix, measured as matrix contraction after release from anchorage [22]. The graphs representing aFPCM compaction (Figure 1A) show that aFPCM containing chitosan derivatives exhibited less compaction compared to control (water). To test whether SWNT itself was inhibitory, a set of aFPCM containing SWNT-PEG (SWNT only) were performed and demonstrated compaction similar to control conditions (Figure 1B) suggesting the GC component of SWNT-GC was the active compaction inhibitor. 

#### 2.1.2. Optical Coherence Tomography (OCT) Imaging Shows Distinctive Morphology and Compaction of aFPCM 

**Figure 2:**
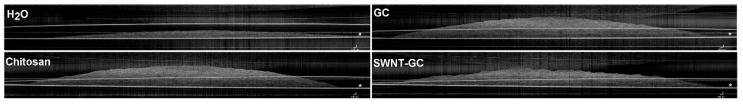
Optical coherence tomography (OCT) imaging comparing morphological differences between aFPCM with and without chitosan derivatives. Matrices were photographed on day 6. Photographed images provided cross-sectional contours of aFPCM at maximum tension generated across each treatment group on day 6. All images were captured before anchorage release for contraction or differentiation assays. The plate line representing the bottom of the aFPCM is beneath the asterisk on the right of each image. Images shown are from one experiment but representative of all reported cell types and experiments.

The 3D organizational aFPCM structure (cross-sectional contour) on or about the day of maximum tension was observed using Optical Coherence Tomography (OCT) imaging (Figure 2). Earlier imaging was limited due to the height of the aFPCM. Maximum aFPCM height occurred near its center, and minimum near-zero height at its peripheral edge, where it was attached to the substratum. The dome height of chitosan derivative-incorporated aFPCM was higher than those of the control (Figure 2, upper left) and SWNT-PEG (not shown) across all tested cell types, demonstrating that presence of chitosan derivatives reduced compaction. 

#### 2.1.3. Tissue Swelling Was Not Observed When Chitosan Derivatives Were Present in The Matrix

**Figure 3:**
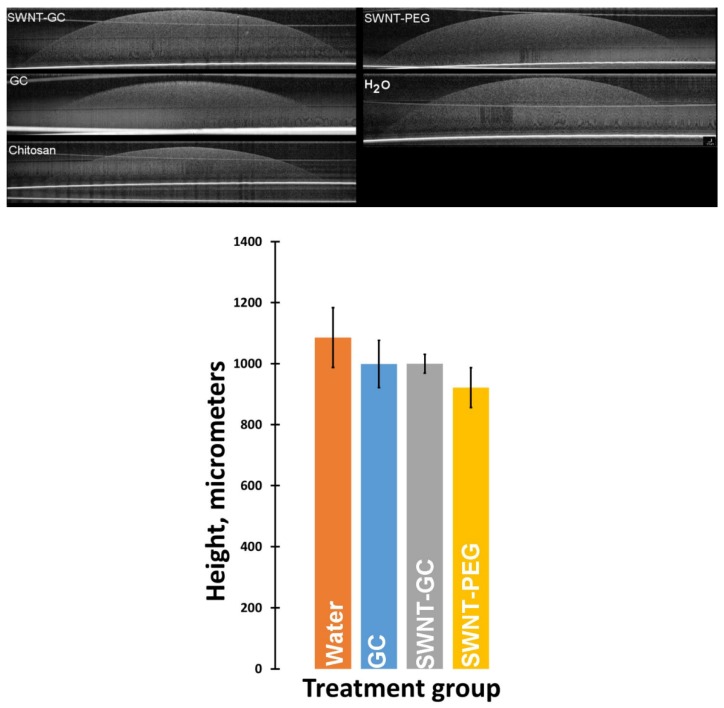
Cell-free matrices with chitosan derivatives demonstrated similar size to water control matrices when viewed with OCT. (**Top**) Matrices were plated using a reduced volume so that the entire height was visible within the depth constraints of the OCT imager. Tissues were imaged 48 h after plating. All matrices were of similar size. (**Bottom**) Bar graph showing the average height of samples after 48 h of culture. Only SWNT-PEG means were SD from control means.

Previous work showed that chitosan incorporation into hydrogels demonstrated a swelling behavior from water absorption [23,24]. We asked whether the compaction inhibition might be due to tissue swelling. To test this, we prepared reduced-volume, cell-free aFPCM with chitosan derivatives against vehicle control (water), and measured aFPCM height over 48 h. In all matrix conditions, the matrix height was similar (Figure 3, top), although the water control matrices were slightly higher (Figure 3, bottom).

### 2.2. Chitosan Derivatives Reduce aFPCM Contraction (Diameter Reduction) When Released from Anchorage

**Figure 4:**
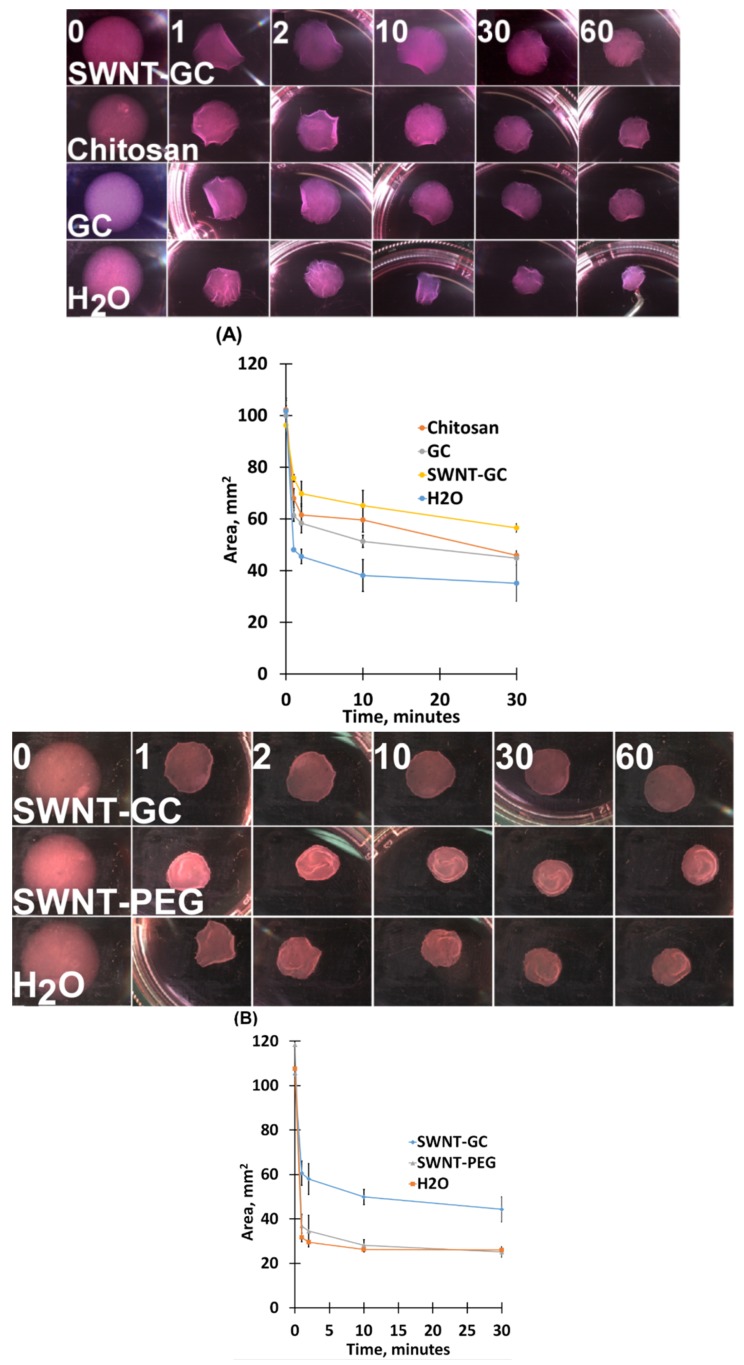
(**A**) Chitosan derivative-containing aFPCM exhibited less contraction compared to controls. Images of aFPCM diameter captured by camera and software showed that chitosan derivative-containing aFPCM, while anchored, had the same diameter as control aFPCM (top, left images), however once released, exhibited a larger diameter (reduced contraction) than control at the measured timepoints. Control means were SD than C and SWNT-GC means for 1-60 min. GC means were SD than control means only at 1, 10, and 60 min. (**B**) SWNT-PEG- but not SWNT-GC-incorporated aFPCM contracted similar to controls. Control means were SD than SWNT-GC means for 1–60 min. SWNT-PEG means were only SD from control means at 0 min. Sample images (top) used to calculate released matrix area changes over the times listed. Data were collected at 0, 1, 2, 10, and 30 min after release from the substratum. 60-min measurement was similar to 30-min measurement (not shown). Additional experiments with other cell types demonstrated similar results.

In aFPCM, compaction and tension generation (inferred by contraction) are correlated [22]. Because chitosan derivatives reduced aFPCM compaction, we predicted that tension generation would be similarly reduced. To test this, we released 6-day cultures of aFPCM from anchorage and measured contraction of the matrix over the following 60 min. Multiple replicate aFPCM from each treatment group were randomly selected to be released and measured. Images of the matrices were captured by camera and software for contraction measurements at 0, 1, 2, 10, 30, and 60 min (Figure 4A, top). The results were then pooled and graphed to demonstrate the biphasic contraction graph characteristic of released aFPCM [22] (Figure 4A, bottom). The aFPCM containing chitosan derivatives contracted less across all different cell types, hence suggesting they generated less tension compared to control matrices. While SWNT-GC reduced contraction, SWNT-PEG did not (Figure 4B).

### 2.3. Chitosan Derivative Presence Was Not Sufficient To Inhibit TGF-β1-Mediated Compaction Increase, Nor Its Correlated Contraction Increase

**Figure 5:**
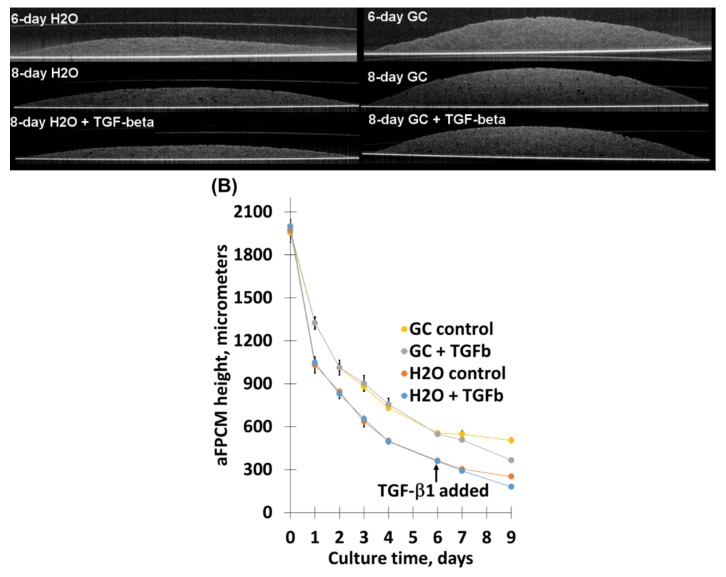
Transforming growth factor beta-1 (TGF-β1) increased compaction in control and chitosan derivative-incorporated aFPCM. Matrices were allowed to approach maximum compaction on day 6, at which time replicate matrices were divided into control and TGF-β1 (5ng/mL)-treated samples. Compaction was measured for 3 days post-treatment. (**A**) OCT showed the TGF-β1-increased compaction in H_2_O-treated (left images) and GC (right images). (**B**) Graphed compaction averages of both H_2_O and GC aFPCM measured by light microscopy. Note that for each of the measured timepoints, GC compaction < H_2_O. The control means were SD than the TGF-β1-treated control means at days 7 and 9. There was no SD between GC control means and TGF-β1-treated GC means, except at day 9. Other chitosan derivatives demonstrated similar compaction changes with TGF-β1 across all tested cell types.

**Figure 6:**
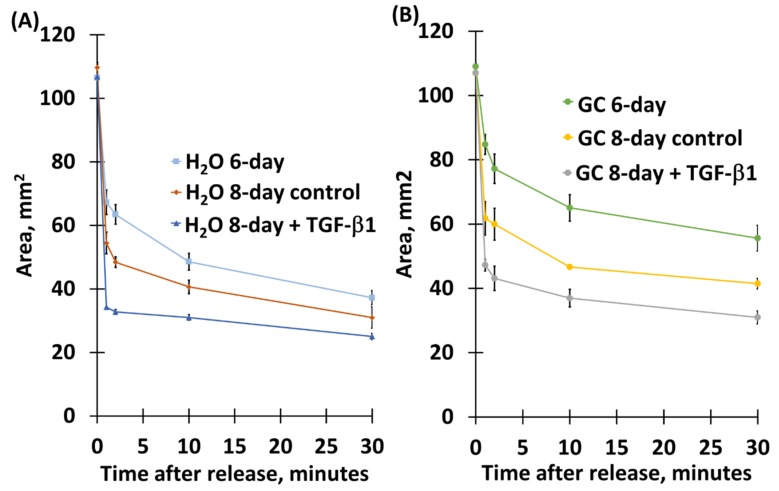
TGF-β1 increased anchorage-released matrix contraction in control and chitosan derivative-incorporated aFPCM. Matrices from each treatment were released and measured at the 6-day timepoint when tension generation was approaching maximum (**A**,**B**) (top line). Remaining matrices were divided into +/- TGF-β1-treatment (5ng/nL) and released and measured 2 or 3 days later. In both H_2_O and GC matrices, 8-day contraction (**A**,**B**) (middle line) was greater than 6-day contraction, and TGF-β1 treated matrices demonstrated the greatest contraction (**A**,**B**) (bottom line). Note that for each of the measured timepoints, GC contraction < H_2_O. The means for the 3 H_2_O groups were SD from each other at 1–30 min, with the exception of the 6-day and 8-day means at 30 min. The means for the 3 GC groups were SD from each other at 1–30 min.

Because TGF-β1 increases collagen matrix reorganization using mechanisms similar to compaction [25,26], we asked whether TGF-β1 would increase compaction and whether chitosan derivative presence would be sufficient to inhibit TGF-β1-increased compaction. To test this, we administered TGF-β1 (5ng/mL) to aFPCM when compaction approached maximum (generally at 6 days of culture), then continued measuring compaction for 3 days. In all cells tested, TGF-β1 increased compaction over continued control-treatment conditions in all aFPCM conditions, measured by OCT (Figure 5A) or standard light microscopy (Figure 5B). As mentioned earlier, compaction is correlated to tension generation. We predicted that chitosan derivative-incorporated aFPCM treated with TGF-β1 would contract to a greater degree than controls. As predicted, TGF-β1 increased contraction of H_2_O matrices (Figure 6A) and GC-incorporated matrices (Figure 6B). Similar results were observed in aFPCM with chitosan and SWNT-GC, in all tested cell types.

### 2.4. Cell Proliferation and Myofibroblast Presence Observed in TGF-β1-Treated Chitosan Derivative-incorporated aFPCM

**Figure 7:**
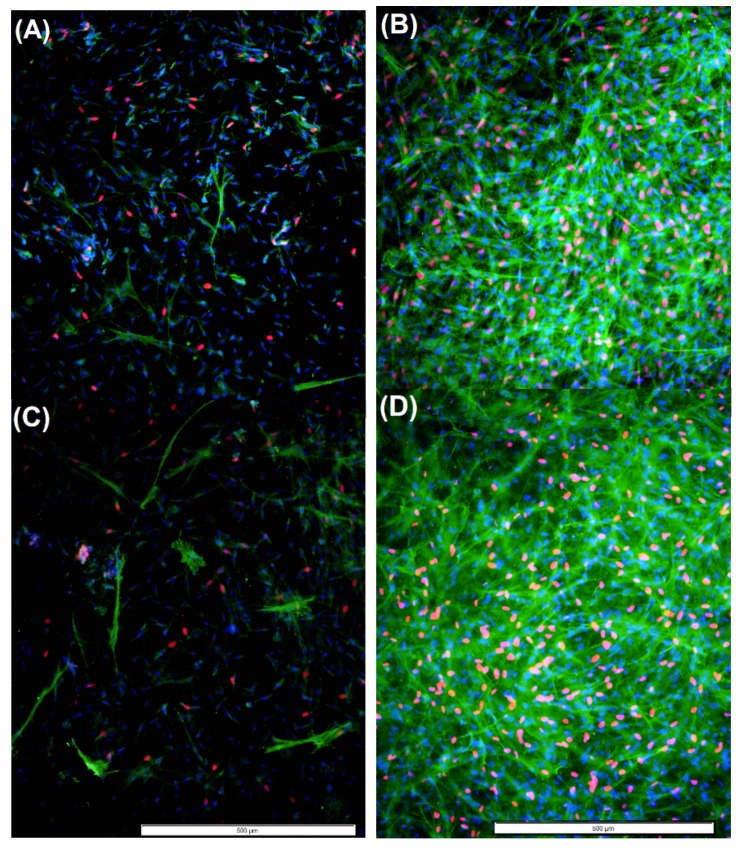
TGF-β1 increases proliferation and myofibroblast differentiation in aFPCM incorporating chitosan derivatives. Immunostaining was used to identify non-proliferating myofibroblasts (green cytoplasmic fibers with blue nuclei), proliferating myofibroblasts (green fibers and pink nuclei), nonproliferating fibroblasts (blue nuclei, and little or no cytoplasmic stain) and proliferating fibroblasts (pink nuclei only). Pink nuclei and green cytoplasmic fibers were abundant in TGF-β1 treated water control aFPCM (**B**) compared to no TGF-β1 treatment (**A**). GC-aFPCM in control conditions (**C**) stained similar to water control aFPCM (**B**), while TGF-β1 treatment increased proliferation and myofibroblasts in GC-aFPCM (**D**) similar to water control (**B**).

We previously noted a decrease in DP139 myofibroblast presence in the presence of GC [27], therefore we tested whether exogenous TGF-β1 administration would be sufficient to induce the myofibroblast phenotype [28,29] in the presence of chitosan derivatives in the extracellular matrix of aFPCM. We employed an immunostaining assay to identify 4 cell types using three stains—proliferating and non-proliferating fibroblasts, and myofibroblasts [3]. Under control conditions near maximum tension, there were only a few myofibroblasts and proliferating cells present in the water control (Figure 7A) and GC (Figure 7C), but each were abundantly present in TGF-β1 treated water control (B) and GC (D). In all conditions, TGF-β1 increased the presence of robust stress fibers containing alpha-smooth muscle actin expression (green cytoplasmic fiber stain). TGF-β1-treatment also increased incorporation of ethynyl deoxyuridine (EdU) nucleotides into click-stained nuclei (pink nuclei). This suggests that TGF-β1 increased proliferation and myofibroblasts in chitosan derivatives and control aFPCM after 3 days of treatment, both of which could account for the increased compaction and contraction.

## 3. Discussion

Our goal was to determine the effects of Chitosan, GC, and SWNT-GC on normal human fibroblasts and Dupuytren’s contracture (DC) cell lines. We hypothesized that they could inhibit compaction, contraction, and differentiation when incorporated into anchored collagen matrices. Our hypothesis was based on GC’s potential to enhance vaccine and anti-tumor responses via inhibiting cancer cell motility and invasion according to past studies [12,13], and published work showing that chitosan could inhibit compaction of a collagen matrix [20]. GC, derived from chitosan, is a nontoxic and water-soluble compound, which allows it to be conjugated with SWNT and permeabilize across the cell membrane [14,15], which may reduce the progression of DC via reversing myofibroblast phenotypes to fibroblasts. Four treatment groups were designed to ensure that GC exhibits the strongest, universal inhibitory effect against proliferation and differentiation of fibroblasts across all cell types. 

We used the stress-relaxed collagen matrix model [30,31], combining the anchored and relaxed collagen matrices, to quantify the mechanical contraction process and study interactions between cells, their ECM, and contractile elements [1]. Results of the matrix height measurements provided supporting and predictive data for the subsequent study of fibroblast-mediated contraction and myofibroblast differentiation [22]. We demonstrated that chitosan-, GC-, and SWNT-GC aFPCM were unable to compact to the same extent as control (water) and SWNT-PEG (SWNT-only) aFPCM across all tested cell lines. This suggested that tension generation was inhibited. OCT imaging was used to determine the morphological structure and surface shape of aFPCM on the day of maximum tension; this data confirmed the light microscopy compaction measurements. Replicate aFPCM were released to measure tension generation, defined as diameter contraction. From Figure 4, measurements of chitosan derivative-containing released aFPCM across different cell lines showed they had a larger diameter compared to control. Therefore, the larger diameter indicates a decrease in contraction or tension generated within the aFPCM. Since our results showed contraction increased as aFPCM height was reduced, our study further confirmed the correlation between compaction and contraction [22]. The OCT system we used was only able to visualize aFPCM after 3 days of compaction when the matrix thickness came within the 1.5 mm depth of field; we developed a reduced volume method to allow us to visualize the cell-free matrices immediately after polymerization (Figure 3). Experiments are underway using this modified protocol to observe early timepoints when cells are becoming accustomed to their newly-synthesized environment [32]. 

To investigate GC mechanisms of inhibiting compaction and contraction, a myofibroblast proliferative assay was performed. Because myofibroblasts play a crucial role in wound contraction, it is likely that the increase in proliferation and differentiation of myofibroblasts correspond to the amount of tension generated within the aFPCM [29,33,34]. Alpha-smooth muscle actin is the protein expressed by differentiated myofibroblasts, which we identified within green cytoplasmic stress fibers of stained tissues; both the protein’s presence and its incorporation into stress fibers is a requirement of myofibroblasts [2]. Previously, we found that GC-containing aFPCM exhibited a reduced number of myofibroblasts in a single tested culture [27]. In the current study we were unable to make a similar observation using different cells (compare Figure 7A to Figure 7C); however, the other cell types tested had a low percentage of myofibroblasts under control conditions relative to the cells previously reported. Other investigators showed a correlation between the intrinsic-stimulated myofibroblast and its increased contractility [34]; a more-robust study using myofibroblasts with higher intrinsic alpha-smooth muscle actin staining characteristics is warranted.

TGF-β1 treatment increased compaction (correlated with proliferation and myofibroblast differentiation staining) in aFPCM that were approaching maximum compaction under control conditions. It is likely that sufficient compaction potential remained to allow TGF-β1 to increase migration-dependent compaction [25,26], because within 24 h the increase was evident. This outpaces the expected time necessary for myofibroblast differentiation, and would require further study to determine specifically whether agonist effect or longer-term myofibroblast differentiation were responsible for the rapid increase. Based on the obtained immunostaining results, chitosan derivatives present in the extracellular matrix were unable to block exogenous myofibroblast differentiation (Figure 7). This suggests a subtle but important difference between endogenous and exogenous myofibroblast stimulation that could be targeted for designing therapies to reduce myofibroblast impact on Dupuytren’s contracture or other fibrotic diseases. Transcription and translation analysis will be required to tease out these differences. Furthermore, a dose-dependent study is needed to determine the optimal dosage of chitosan derivatives to elicit their maximum effect. 

## 4. Materials and Methods 

### 4.1. Human Cell Cultures 

Human dermal fibroblasts (HDF00703, HDF01035, HDF03761, purchased from LifeLine Cell Technology, Frederick, MD, USA) and Dupuytren’s contracture (DC) fibroblasts isolated from the diseased nodule (DP139, DP 141a, and DP147; gift of James J. Tomasek, University of Oklahoma Health Science Center, Oklahoma City, OK, USA) were cultured under standard conditions using media containing Dulbecco’s Modified Eagle Media (DMEM) and high glucose (Gibco; Fisher Scientific, Waltham, MA, USA) supplemented with 5% fetal bovine serum (Atlanta Biologicals, Atlanta, GA, USA), 2 mM glutamine, and 1% antibiotic-antimycotic (Sigma Chemical Company, ST. Louis, MO, USA). Cells were kept in log-phase growth and used when 90% confluent. The use of human cells and other methods described above were in compliance with the University of Central Oklahoma Institutional Review Board. The Institutional Review Board-approved study was University of Central Oklahoma # 08077. DC cells used in this study were collected from the nodules of stage-4 DC patients (those having the highest degree of flexion).

### 4.2. Preparation of aFPCM 

#### Anchored Fibroblast-Populated Collagen Matrix (aFPCM) Assay

The aFPCM represents an appropriate three-dimensional model to study wound healing and fibrosis in vitro. Matrices were set up as previously described [29,33]. For this study, six matrices were set up per treatment group. Fibroblasts (1.25 × 10^5^ cells/mL final concentration) were combined with type I bovine collagen (1 mg/mL; Gibco) or rat tail collagen (Corning) equilibrated with NaOH and NaHCO_3_. In experimental lattices, 10% of the media volume was replaced with either chitosan (Sigma, St. Louis, MO, USA), *N*-dihydrogalactochitosan (GC) (Immunophotonics, St. Louis, MO, USA), GC conjugated with single-walled carbon nanotubes (SWNT-GC), or SWNT-polyethylene glycol (SWNT-PEG); all were dissolved in water. In control matrices, water replaced 10% of the media volume. The solution was mixed for two to four minutes. Then, 140 µL of the mixture was plated onto a prewarmed tissue culture dish (Techno Plastic Products (TPP); Midwest Scientific, St. Louis, MO, USA). For cell-free matrix studies, 90 µL of the original 140 µL were removed to lower the matrix height. The aFPCM were placed in a 37 °C incubator for polymerization for 1 h before immersing in 2 mL culture media. Every other day, for 6 days, the media was changed by replacing 1 mL of old media with new media to ensure appropriate cell functions. For myofibroblast induction experiments, on day 6 growth media was replaced with growth media including 5ng/mL transforming growth factor beta-1 (TGF-β1; Sigma Aldrich, St. Louis, MO, USA) or vehicle control media, and culture continued for 2–3 days.

### 4.3. Compaction Measurements of aFPCM Assay 

#### 4.3.1. The aFPCM Height Measurement using Zeiss Primovert Microscope 

The aFPCM compaction was defined as matrix height reduction caused by tension generated within the matrix. Measurements were analyzed as previously described [22].The height of the aFPCM was measured once every day during the 6-day period, beginning one hour after polymerization, when growth media was added, and aFPCM were returned to incubation at 37 °C. Compaction was measured using the Zeiss Primovert inverted-light microscope. With an aFPCM placed on the microscope stage, the phase slider was adjusted to “20×” for maximum light availability. The matrix was moved until the small circle of light was centered over the apex of the matrix. Fine fibers at the apex of the aFPCM were identified as the focus start point, where the coarse and fine focus knobs were zeroed. The user fine-focused through the matrix until identifying the aFPCM underside, where flattened cells were observed, while counting the units on the fine focus wheel. The quantified number of measured units was recorded. Measurements were hand-written onto a lab log book and transferred to an Excel spreadsheet. The aFPCM height in micrometers was determined by using the formula = (cell/640) × 1000, where 640 equals the microscope units needed to focus through a 1mm-thick microscope slide. A scatterplot line graph was established using the average +/− standard deviation generated using Excel formulae AVERAGE and STD. Replicates before TGF-β1 treatment were n = 6 or greater, while after treatment n = 3.

#### 4.3.2. Optical Coherence Tomography (OCT) Imaging on aFPCM 

The OCT imaging system (Thorlabs, Newton, NJ) provides micrometer resolution and millimeter penetration depth in biomedical imaging [27]. After manual aFPCM height measurements on day 6, images of matrix contour based on the location and time of reflected light were captured by OCT. The consequent number of units measured was recorded. Images of aFPCM photographed on day 6 exhibited maximum tension generated and were used to predict the effect of GC on contraction and differentiation. After OCT imaging, aFPCM were prepared for release and contraction measurements.

### 4.4. Released aFPCM Contraction Assay

On day 6, the aFPCM approached maximum tension generation. Three matrices from each treatment group were photographed using a dissecting light stereoscope (Olympus SZ61), then released mechanically from the substratum. To begin, the edges of the aFPCM were lifted by a dissecting needle then media was gently pipetted under the lifted edges to detach the remainder [35]. Images were collected after release at times 1, 2, 10, 30, and 60 min via a digital camera (Insight; Diagnostic Instruments Incorporated, Sterling Heights, MI, USA) and SPOT software (Diagnostic Instruments Incorporated, Sterling Heights, MI, USA). Matrices remained in the culture incubator when not being photographed. Measurements of matrix area were analyzed using ImageJ software (National Institutes of Health, Washington, DC, USA).

### 4.5. Myofibroblast Proliferative Assay

Proliferation and myofibroblast differentiation are important modifiers of collagen matrix contraction [29,34]. To test for proliferation, aFPCM were incubated for 3 h in the presence of 10 μM ethynyl deoxyuridine (EdU; a modified nucleotide; BaseClick; Millipore Sigma, ST. Louis, MO, USA) and half-volume of fresh media plus treatments prior to fixation. To properly evaluate the nature of myofibroblasts and their proliferative activity on a three-dimensional level, control and treatment aFPCM were fixed with 4% paraformaldehyde and fluorescently stained with anti-α-smooth muscle actin (α-SMA, clone 1A4), Baseclick-EdU 594, and DAPI (4′,6-diamidino-2-phenylindole; Millipore Sigma). Previously prepared aFPCM were separated into halves by use of a scalpel. Halves were carefully transferred to microcentrifuge tubes containing phosphate-buffered saline. Preceding the fluorescent staining, tissue was treated with -20 °C methanol for 10 min, which precipitated the proteins and opened the cellular membranes. Shortly thereafter, the tissues were submerged with 100 µL goat serum/PBS (1:10) followed by the primary antibody; alpha smooth muscle actin/PBS azide (1:500) overnight at 4 °C. After PBS washes, goat anti-mouse IgG Alexa 488/PBS azide (1:200), working as the secondary antibody, was introduced to the lattices for 45 min. Detection of proliferative activity within cells was achieved through Baseclick-Edu 594 stain for 30 min. The DAPI/PBS azide (2:1000) stain was then administered to the lattices, wherein all nucleated cells absorbed the highly specific blue-fluorescent dye excited by 405 nm laser light.

Following the fixation and staining procedure, the matrices were prepared for analysis under fluorescent microscopy. Prior to mounting the matrices on microscope slides, 80% glycerol in PBS at 4 °C was applied to each microcentrifuge tube containing the matrices for 10 min. Tissues were teased out of the microcentrifuge tube with forceps and deposited on the microscope slides containing 40 µL of previously placed glycerol/PBS. Then, 22 mm^2^ coverslips placed over the flattened tissues were sealed with coverslip sealant and allowed to dry before microscopy or storage at −20 °C.

### 4.6. Imaging and Analysis

Through combination of CelSens software (Olympus Corporation of the Americas, Center Valley, PA, USA) and the IX-71 Olympus inverted fluorescent scope with DP 72 high-speed camera (Olympus Corporation of the Americas, Center Valley, PA, USA), stained aFPCM samples were analyzed and imaged. Objectives used include: 10× UPLSAPO numerical aperture 0.40, LUCPLFLN 20× numerical aperture 0.45 and LUCPLFLN 40× numerical aperture 0.60. Images were captured individually with the camera using specific wavelength filters pertaining to each stain. Images were overlapped using the “combine color images” and “burn in info” commands within the CelSens program.

### 4.7. Calculations/Analysis

All data analyzed in this study are presented as the mean +/− standard deviation (SD). Statistical Analysis Software (SAS) Version 9.4 was used for statistical analysis. Analysis of variance was used to determine significant differences (*p* < 0.05) between means. Pairwise comparisons were performed using Tukey’s multiple comparison tests. The aFPCM compaction was measured using a formula in Microsoft Excel (= (cell value/640) × 1000), which changes the number of fine-focus microscope units into micrometers for all measurements, as previously calibrated [22].

## 5. Conclusions

This study successfully identified the effects of chitosan derivates using an in vitro model of fibrotic diseases such as DC. Chitosan derivatives incorporated directly into the aFPCM inhibited compaction and contraction. OCT images provided insight for the effects of chitosan derivatives on aFPCM structure and organization. Our results showed the correlation between a reduced compaction in chitosan aFPCM and a decrease in tension generation via a decrease in fibroblast differentiation and myofibroblast presence. We showed that although tension and compaction were reduced with chitosan derivatives, sufficient tension was present to allow myofibroblast differentiation in the presence of TGF-β1. Therefore, further investigation of chitosan derivative dose-dependent in vitro response is needed.
